# Prehospital use of plasma in traumatic hemorrhage (The PUPTH Trial): study protocol for a randomised controlled trial

**DOI:** 10.1186/s13063-015-0844-5

**Published:** 2015-07-30

**Authors:** Penny S. Reynolds, Mary Jane Michael, Emily D. Cochran, Jacob A. Wegelin, Bruce D. Spiess

**Affiliations:** Department of Anesthesiology, Virginia Commonwealth University Medical Center, Richmond, VA USA; Department of Biostatistics, Virginia Commonwealth University, Richmond, VA USA

**Keywords:** Bleeding, coagulopathy, inflammatory markers, INR, massive hemorrhage, prehospital, shock, thawed plasma, TRALI, transfusion, trauma

## Abstract

**Background:**

Severe traumatic injury and haemorrhagic shock are frequently associated with disruptions of coagulation function (such as trauma-induced coagulopathy TIC) and activation of inflammatory cascades. These pathologies may be exacerbated by current standard of care resuscitation protocols. Observational studies suggest early administration of plasma to severely-injured haemorrhaging patients may correct TIC, minimise inflammation, and improve survival. The proposed randomised clinical trial will evaluate the clinical effectiveness of pre-hospital plasma administration compared with standard- of-care crystalloid resuscitation in severely-injured patients with major traumatic haemorrhage.

**Methods/design:**

This is a prospective, randomized, open-label, non-blinded trial to determine the effect of pre-hospital administration of thawed plasma (TP) on mortality, morbidity, transfusion requirements, coagulation, and inflammatory response in severely-injured bleeding trauma patients. Two hundred and ten eligible adult trauma patients will be randomised to receive either two units of plasma, to be administered in-field, *vs* standard of care normal saline (NS). Main analyses will compare subjects allocated to TP to those allocated to NS, on an intention-to-treat basis. Primary outcome measure is all-cause 30-day mortality. Secondary outcome measures include coagulation and lipidomic/pro-inflammatory marker responses, volume of resuscitation fluids (crystalloid, colloid) and blood products administered, and major hospital outcomes (e.g. incidence of MSOF, length of ICU stay, length of hospital stay).

**Discussion:**

This study is part of a US Department of Defense (DoD)-funded multi-institutional investigation, conducted independently of, but in parallel with, the University of Pittsburgh and University of Denver. Demonstration of significant reductions in mortality and coagulopathic/inflammatory-related morbidities as a result of pre-hospital plasma administration would be of considerable clinical importance for the management of haemorrhagic shock in both civilian and military populations.

**Trial registration:**

ClinicalTrials.gov: NCT02303964 on 28 November 2014

## Background

Following severe traumatic injury, the immediate care priorities are control of bleeding and correction of the hypovolemia and tissue hypoperfusion that result from excessive blood loss and tissue injury. Massive hemorrhage (MH) requires surgical intervention for definitive hemostasis. However, trauma-related hemorrhagic shock is also a disease of ischemic cellular injury and death, resulting from coagulation disruption and inflammatory precursor activation [7, 15, 23]. Approximately 25 % of severely injured patients present with severely abnormal coagulation changes occurring within very few hours of injury [22]. This so-called trauma-induced coagulopathy (TIC) is associated with 4-fold increase in mortality, increased rates of late death from sepsis and multiple organ failure (MOF), and worsened outcomes from traumatic brain injury (TBI) [4, 5, 13]. Ischemic cellular injury resulting from uncorrected, persistent, and systemic hypoperfusion eventually leads to systemic inflammatory response syndrome and irreversible multi-system organ failure (MSOF). MSOF is a major cause of late mortality and morbidity following severe trauma, with a mortality rate of 50 % to 80 % [20].

To minimize irreversible cell damage and death, it follows that recognition of MH should occur as soon as possible after injury and as far upstream as possible from initiation of coagulopathy/inflammatory cascades. Unfortunately early recognition may not be easily achieved, especially if bleeding is primarily internal. In practice, the extent of hemorrhage and shock during the initial assessment stage is based on vital signs, primarily blood pressure, pulse rate, palpable radial pulses, and mentation [1]. However these are extremely variable, non-specific, and very poor indicators of perfusion status [29]. Therefore choice of first-line resuscitation fluids will be essential for preempting some of the negative outcomes of unrecognized MH and shock.

There is increased interest in reviving prehospital use of plasma transfusions for severely injured hemorrhaging patients, especially if time to definitive care is long. Observational data suggest improved 6-hr outcomes but no overall survival advantage with prehospital blood product administration [17]. Better patient 30-d survival was associated with earlier (<4 hr) in-hospital administration of relatively high ratios of plasma to packed red blood cells (pRBC) in both civilian [10, 18] and military [3] trauma populations; meta-analysis supports early use of plasma but does not conclude that there are additional survival benefits of 1:1 over 1:2 plasma:pRBC transfusion ratios [2] In contrast, plasma transfusions have been associated with increased morbidity and mortality in other studies of trauma [21] and non-MH patients [24, 26, 27]. However, interpretations of data from these studies may be confounded by lack of standardization in hospital resuscitation protocols, changes in transfusion practice over the past decade, confounding-by-indication bias [19] and survivor bias [16].

At present there are no data either from systematic reviews [11] or from completed, prospective, randomized controlled clinical trials [19] to assess the efficacy of plasma transfusion alone. Further, there are no rigorous definitions of how early infusion must occur relative to injury in order to demonstrate benefit. This study will be one of three prehospital trials conducted in parallel that explicitly examines the effect of prehospital plasma transfusion on mortality and morbidity in civilian trauma patient populations.

## Methods/Design

### Study design

**Fig. 1:**
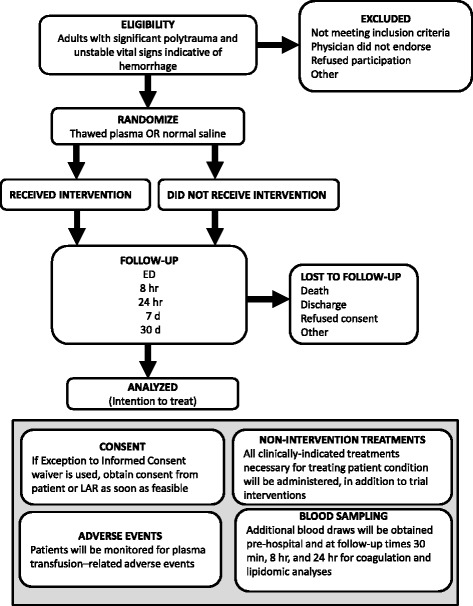
Consolidated Standards of Reporting Trials (CONSORT) flow diagram of phase progress for the Virginia Commonwealth University (VCU) plasma trial (PUPTH), a parallel randomized trial of prehospital administration of plasma vs standard-of-care normal saline in a trauma population. *ED* Emergency Department

This study is a prospective, open-label, non-blinded, randomized clinical trial to quantify the effects of early prehospital administration of plasma on death, coagulation function, transfusion requirements, and other relevant clinical outcomes for seriously-injured trauma patients with massive bleeding. Trial flow per the Consolidated Standards of Reporting Trials (CONSORT) guidelines [25] is shown in Fig. 1.

### Ethical oversight

The study was approved by the Virginia Commonwealth University Institutional Review Board (VCU-IRB Protocol Record HM14813), Food and Drug Administration (FDA) (IND no. 15910); and the US Department of Defense (HRPO Log Number A-17160). The study was listed with the Clinical Trials register on 28 November 2014 (ClinicalTrials.gov number NCT02303964).

### Study population

The study population consists of adult, physiologically unstable, polytrauma patients transported to Virginia Commonwealth University Medical Center (VCUMC), a Level I trauma center. The catchment area is Henrico County and City of Richmond, Virginia. Henrico Fire EMS system (HFD) serves a population of 325,000 with over 30,000 transports per year; the city of Richmond EMS system (Richmond Ambulance Authority, RAA) serves a metro area of 222,000 with over 27,000 transports per year.

#### Inclusion criteria

Patients eligible for this study include all adult (≥18 years) patients of either sex, with blunt and/or penetrating trauma with multi-system injuries and unstable vital signs (systolic blood pressure (SBP) ≤70 mmHg, or SBP 70–90 mmHg with heart rate (HR) ≥108 beats per minute (bpm)), indicative of severe ongoing hemorrhage.

Exclusion criteria are: (1) presence of an opt-out wrist band, wallet card, Medical Alert jewelry, bracelet, or similar identifier to indicate Jehovah’s Witness or similar patient group objecting to transfusion of blood and/or blood products; (2) refusal of consent by patient or legally appointed representative (LAR); (3) language barrier (non-English or non-Spanish speaking); (4) not expected to survive transport to VCUMC (based on extreme severity of injuries, on-scene cardiac arrest, and/or cardiopulmonary resuscitation (CPR) prior to randomization); (5) documented do not resuscitate (DNR) order; (6) isolated penetrating head trauma; (7) burns to >20 % of the body surface; (8) pregnancy; (9) prisoner; (10) if patient transport has proceeded before arrival at the scene by the emergency medical services (EMS) supervisor with plasma, and (11) failure of the EMS to obtain intravenous (IV) access.

#### Patient drop-out

Patients may refuse consent and leave the study at any time for any reason. Subjects can be withdrawn from the study emergently by the attending study physician if they develop signs and symptoms of acute transfusion reaction.

### Interventions and controls

Patients will be randomized prior to arrival at the Emergency Department (ED) to receive either thawed fresh-frozen plasma (TP) or standard-of-care normal saline (NS) during prehospital transport. Patients randomized to the TP arm will receive up to two units of pre-thawed A+ plasma. The control intervention is standard-of-care NS administered IV/intra-osseous (IO) per prehospital (ODEMSA) protocol to maintain SBP at 90–100 mmHg.

The VCUMC Blood Bank will provide plasma to EMS supervisors. Use of low-titer anti-B (<1:100) A+, rather than AB (universal donor), plasma is justified on the grounds of the scarcity of AB plasma, and because of the extremely small risk of hemolytic reaction associated with administering A+ to patients who are not blood group compatible (incidence 0.1–1 %) [9]. TP will be maintained in dedicated refrigeration units in the EMS supervisor vehicle; unused TP will be exchanged within 24 hr to the VCUMC Blood Bank.

### Randomization

Eligible patients will be randomly assigned to receive either TP or NS prior to ED arrival. Randomization codes will be generated using complete randomization; code sequence is secured by trial statisticians, and held at the VCUMC Transfer Call Center in sealed opaque envelopes. Patients will be screened for eligibility by on-scene prehospital providers. Endorsement for plasma administration will be given by the study physician on call. At this point the patient is considered enrolled into the study. Transfer Centre personnel will then open the next envelope in sequence and advise EMS as to patient allocation.

### Study outcomes

The primary outcome of this study will be all-cause 30-day mortality. Secondary outcomes are: (1) hemodynamics (specifically SBP, mean arterial pressure (MAP), and HR); (2) cumulative volume of resuscitation fluids and blood products utilized at 8 and 24 hr from injury (volumes of crystalloid and/or colloid, units of red blood cells, units of plasma, doses of platelets, doses of cryoprecipitate); (3) hematology (hemoglobin (Hb) and hematocrit (Hct)) and blood biochemistry (pH, lactate, base deficit, and bicarbonate); (4) coagulation function (thromboelastography (TEG), fibrinogen, FV, FVIII, prothrombin time (PT), activated partial prothrombin time (aPTT), von Willebrand’s factor, D-dimer, PFA-100, and platelet count); (5) lipidomic profile (arachidonic acid, eicosanoid, and prostacyclin expression); (6) hospital outcomes on days 7 and 30 (rates of MSOF, rates of acute renal failure (ARF), number of surgeries, number of infections, duration of mechanical ventilation, length of ICU stay, and length of hospital stay).

### Other data

Patient demographics (age, sex, height, weight, past medical history, comorbidities, medications), mechanism of injury, injury severity assessments—Glasgow coma score (GCS), injury severity score (ISS), sequential organ failure assessment (SOFA), and acute physiology and chronic health evaluation (APACHE IV), routine vital signs and laboratory data, hospital-care-mandated medications and interventions (e.g., intubation, mechanical ventilation, chest thoracotomy, and needle decompression, etc.)—will be collected from individual medical records as they become available after patient admission.

### Timelines

Follow up of patients enrolled in the trial ends at death or 30 days post-randomization, whichever occurs first. Patients discharged before 30 days will be followed up via telephone by designated study personnel. Blood samples for coagulation function and lipidomic profiling will be collected from each patient at four pre-specified time points: prehospital (close to point of injury, and before prehospital resuscitation), in the ED (approximately 30 min from hospital admission), and at 8 and 24 hr from admission.

### Estimated event rate and sample size calculations

VCU Trauma Center registry data suggest that 30-day mortality under the standard-of-care protocol is between 16.4 % and 29.2 %; this is similar to the estimated 26 % mortality for the control arm of the Resuscitation Outcomes Consortium (ROC) prehospital hypertonic saline trial [6]. Assuming 24 % mortality in the control group, a sample size of 105 per arm will achieve 80 % power to detect a clinically significant reduction in mortality of 60 % (9.6 % mortality) in the plasma group [12].

### Disclosure, consent, and confidentiality protection

#### Community consultation and public disclosure procedures

Pre-trial public disclosure (required by 21 CFR 50.24) was completed in 2013–2014. Information was disseminated by community meetings, web-based and social media outlets, paid advertisements, and media news releases to TV, radio, and newspapers. Public disclosure included a summary of investigational plans, background information about the study, a synopsis of the protocol and study design, risks and benefits of plasma versus the standard crystalloid fluid, subject selection, exception from informed consent requirements, procedures for contacting LARs, and procedures for declining participation in the study. Following study termination, findings of the clinical trial will be disseminated to the community by media news releases, the trial website (http://www.cctr.vcu.edu/news/feature/plasma.html), and relevant patient organizations.

#### Consent procedures

Significant multi-system trauma with either overt or occult massive hemorrhage is a life-threatening emergency. As treatment must be initiated as early as possible, and severely injured patients may be either unable or not competent to give fully informed consent, this trial will be conducted under the Exception from Informed Consent Requirement (21 CFR 50.24). However all reasonable attempts to impart trial information and obtain consent will be made, depending on the urgency of the immediate medical situation, the mental status of the patient, and the availability of an LAR or next of kin.

After patient admission, and if the patient is unable to consent, attempts to contact the LAR or adult next of kin will be initiated (1) no later than 30 min post-ED arrival, then (2) every 30 min for 2 hr, (3) at least three more times for the remainder of the first 24 h, (4) at least twice a day through the end of day 7, and (5) at least once per week through the end of day 30, death, or discharge.

Confidentiality will be maintained by securing paper-based records in a central secure location and electronic records by access controls and encryption, both to be accessed only by authorized study personnel. All records will be de-identified and data coded with the key stored in a separate secure location.

### Post-randomization treatment

All clinically indicated treatments (including blood product and fluid administration) will be given as appropriate per attending physician discretion. Standard of care for polytrauma patients with MH includes administration of TP plus blood products and/or crystalloid and colloid shortly after reaching VCUMC ED, unless otherwise indicated. In addition to administration of trial resuscitation treatments (TP or NS), trial-specific interventions will include blood sampling for coagulation function and lipidomic determinations; Approximately 20 mL of blood will be obtained from each patient at four time points.

### Trial management

#### Stopping rules

It is expected that the trial will terminate when the intended sample size has been achieved. However, the trial will be stopped prior to completion if: (1) the intervention is associated with adverse effects that call into question the safety of the test intervention; (2) problems with study recruitment or retention will significantly impact the ability to evaluate the study endpoints; (3) any new information that becomes available during the trial necessitates discontinuation of the trial. The Principal Investigator (PI) will include an assessment of futility in the annual progress report to the FDA (using statistical means such as predictive probability, if appropriate), and will consult with the Data and Safety Monitoring Board (DSMB) to assess the impact of significant data loss due to problems in recruitment, retention, or data collection.

#### Trial Steering and Trial Management Committees

The Trial Management Committee consists of the Trial Steering Committee members plus a Trial Data Manager, and Trial Financial Administrator (Table 1). Responsibilities include (but are not limited to: (1) maintenance of trial records; (2) confirmation of regulatory approval compliance before the start of the trial; (3) provision of trial-specific training; (4) provision of specialized trial materials; (5) data management and security; (6) regular communication with collaborators as to study progress; (7) safety reporting; (8) statistical analyses, and (9) publication and dissemination of trial results.

**Table 1:** Members of the Prehospital use of plasma in traumatic hemorrhage (PUPTH) Trial Steering and Management Committees

Name	Affiliation	Role
Bruce D Spiess, MD	Department of Anesthesiology	Principal Investigator
Mary-Jane Michael, RN, MS	Department of Anesthesiology	Trial Coordinator
Chris Hogan, MD	Department of Emergency Medicine and Trauma Surgery	Clinical expert
Joseph P Ornato, MD	Department of Emergency Medicine	Clinical expert
Ron Daniel, MD	Department of Anesthesiology	Trial Safety Officer
Penny S Reynolds, PhD	Department of Anesthesiology	Trial expert
Emily Cochran, RN, MS	Department of Anesthesiology	Trial expert
Jacob Wegelin, PhD	Department of Biostatistics	Trial Statistician
Brian Bush, PhD	Department of Biostatistics	Data management, IT
Carolyn Jeter	Department of Anesthesiology	Financial administration
William Aiken, Capt.	Henrico Department of Fire	EMS
Wayne Barbour	Richmond Ambulance Authority	EMS
Solomon Luckett	Richmond Ambulance Authority	EMS

#### Publication and dissemination of results

The trial protocol and results will be published in peer-reviewed journals. All publications will follow the CONSORT statement [25]. Credit for the study will be assigned to collaborators who have actually performed designated work. Links to published data will be provided through the ClinicalTrials.gov website. The results of the trial will be reported first to trial collaborators. Dissemination of results to patients will take place via the media, relevant patient organizations, and the trial website (http://www.cctr.vcu.edu/news/feature/plasma.html).

### Safety and adherence oversight

#### Data Safety Monitoring Board (DSMB) oversight

An independent DSMB has been appointed to oversee safety monitoring and trial data (Table 2). DSMB duties and responsibilities are outlined in the DSMB Charter tailored specifically for this trial and outlined in accordance with charter guidelines proposed by the DAMOCLES Study Group [8]. The DSMB will review cumulative data at regular intervals over the course of trial, and advise the TSC apropos the safety of both currently enrolled subjects and those to be recruited. The trial may be terminated early by the Trial Steering Committee and Trial Safety Officer on recommendation from the DSMB. Study stopping criteria include futility (significantly increased risk of serious adverse effects in one of the treatment groups based on prespecified stopping boundaries for the primary outcome) and lack of feasibility (e.g., extremely low patient recruitment, excessive patient dropout, etc.).

**Table 2:** Members of the independent Data Safety and Monitoring Committee

Name	Affiliation	Expertise
Neil Blumberg, MD	University of Rochester Medical Center, New York	Clinical expert, DSMB chair
Kevin R Ward, MD	University of Michigan School of Medicine, Michigan	Clinical expert
Rao Ivatury, MD	Virginia Commonwealth University Medical Center, Richmond, Virginia	Clinical expert
Roy Sabo, PhD	Virginia Commonwealth University School of Medicine, Virginia	Independent statistician

#### Adverse events

Adverse transfusion-related events are extremely rare in the USA (0.24 % in 2011). Clinical events that are considered transfusion reactions include: fever (body temperature change >2 °C), urticaria, pruritis, flushing, hypotension, respiratory distress, bronchospasm, transfusion-related acute lung injury (TRALI), and transfusion-associated circulatory overload (TACO) [28].

Study progress and safety will be reviewed weekly by the Safety Oversight Officer. Assessment and reporting progress reports will be provided to DSMB; these reports will include numbers for patient recruitment, retention, attrition, and adverse events (AE) and serious adverse events (SAE). All reported AE will be assessed for relationship to the study intervention, reviewed as to treatment arm and further classified by severity (mild, moderate, severe, life threatening, or disabling), probability (expected vs unexpected), actions taken, and resolution, and will be reported to VCU-IRB.

#### Protocol adherence

Data on adherence to the study protocol will be collected weekly by research staff and reviewed quarterly by the PI and the Safety Officer. Weekly adherence will be evaluated by EMS supervisors and the Trial Safety Officer by monitoring four adherence criteria for each enrolled patient: (1) patient met inclusion criteria, (2) patient was appropriately randomized, (3) patient received appropriate fluid, and (4) patient had prehospital blood samples drawn. Scene and transport times are obtained routinely in both EMS systems, and if feasible will be compared to non‐protocol case times to assess if protocol adherence results in unusual delays in time to definitive care. If adherence falls below the suggested rate of 80 %, which might affect study integrity in terms of testing the primary hypotheses, the Safety Officer will suggest a conference call for study investigators and EMS personnel to discuss methods for improving adherence. Adherence and deviations from the study standard operating procedure (SOP) will be reported to the Trial Coordinator and the VCU-IRB.

### Data management

The VCU Biostatistics Data Coordinating Group created standard data entry procedures and systems for web-based data management during the community consultation phase of the PUPTH trial (completed in 2013), and will continue to oversee data collection, data entry, and data quality assurance. This trial will utilize the REDCap platform for data collection and storage [14]. Staff will coordinate with the University Computer Center to back up and archive data daily, and generate reports as required by the investigators.

### Data analyses

Primary analyses will compare primary and secondary outcomes of patients assigned to either TP or NS on an intention-to-treat basis. Definitive analyses will occur after the 30-day observation period for all survivors in each arm has been completed. Data will be assessed on de-identified data by statisticians and data analysts blinded to patient randomization and allocation category. Appropriate analysis strategies to reduce effects of survival and time-dependencies in post-admission treatments will be applied as necessary.

Planned subgroup analyses will be stratified on: (1) primary mechanism of injury (blunt or penetrating); (2) injury severity (severe ISS 16–24, critical ISS ≥ 25); (3) presence or absence of traumatic brain injury, defined by GCS categories (severe 3–8, moderate 9–12, mild/absent 13–15) or by abbreviated injury score-head (AIS-H) categories (yes ≥3; no <3); (4) presence or absence of severe shock/tissue hypoperfusion (yes defined by base deficit ≥6 and lactate ≥3 mmol/L; no otherwise). Interaction tests will be used to examine the effect of the intervention (if any) across subgroups.

Interim analyses: there will be three planned analyses. Two interim analyses will be performed following accrual of 70 patients (35 per arm) and 140 patients (70 per arm), respectively. Interim and final data will be submitted to the DSMB for review; stopping criteria are based on safety data (see *Stopping rules*). Records will be assessed for data quality, including completeness (thereby encouraging collection of high-quality data), rates of recruitment and losses to follow up, and rates of protocol compliance.

## Discussion

Benefit of early in-hospital use of TP to correct trauma-related coagulation dysfunction is weakly supported by several observational studies [2], but unsupported or contradicted by other studies [21, 27]. However, confounding bias inherent to such studies, coupled with elastic definitions of what constitutes early in the critical care timeline, means that these data are not particularly informative with respect to the immediate care of critically injured patients in hemorrhagic shock. This study is designed to assess the efficacy of prehospital plasma resuscitation on clinically relevant endpoints of mortality and short-term coagulation and inflammatory response.

There are several limitations to this study: TP infusion cannot be blinded to prehospital providers (although subsequent healthcare professionals may not necessarily receive this information), variable scene–hospital transport times of 6–30 min, so that not all patients allocated to the TP arm will receive the full complement of two units of TP before hospital arrival, and the complexity of patient management in the field, which means that the prehospital period will be most vulnerable to missing data.

This trial, coupled with those at Pittsburgh and Denver sister institutions, will be among the first to test the hypothesis that prehospital plasma administration to severely injured patients is effective in reducing trauma-related mortality and coagulopathy. Resulting data will be of considerable clinical importance for informing the management of hemorrhagic shock in both civilian and military populations.

## Trial status

The trial has begun enrolling patients. The expected average enrollment rate is one patient per week until 2016.

## Abbreviations

AABB: American Association of Blood Banks
AIS-H: abbreviated injury score-head
APACHE IV: acute physiology and chronic health evaluation IV score
aPTT: activated partial prothrombin time
bpm: beats per minute
DNR: do not resuscitate
DoD: Department of Defense
DSMB: data safety monitoring board
ED: Emergency Department
EMS: emergency medical services
FDA: Food and Drug Administration
FFP: fresh-frozen plasma
GCS: Glascow coma score
Hct: hematocrit
HFD: Henrico Department of Fire
Hb: hemoglobin
HR: heart rate
HS: hypertonic saline
IND: Investigational New Drug
ISS: injury severity score
LAR: legally authorized representative (according to Code of Virginia Definition 32.1-162.16)
MAP: mean arterial pressure
MH: massive hemorrhage
MSOF: multi-system organ failure
NIH: National Institute of Health
NS: normal saline (0.9 % NaCl solution)
PI: Principal Investigator
PT: prothrombin time
PUPTH: Prehospital use of plasma in traumatic hemorrhage
RAA: Richmond Ambulance Authority
RBC: red blood cells
ROC: Resuscitation Outcomes Consortium
SBP: systolic blood pressure
SC: Study Coordinator
SOFA: sequential organ failure assessment
SOP: standard operating procedure
TACO: transfusion-acquired circulatory overload
TBI: traumatic brain injury
TCCC: Tactical Combat Casualty Care
TEG: thromboelastography
TIC: trauma-induced coagulopathy
TRALI: transfusion-related acute lung injury
VCUMC: Virginia Commonwealth University Medical Center

## References

[1] ATLS (1997). Committee on Trauma, American College of Surgeons. Advanced Trauma Life Support Program for Doctors.

[2] Bhangu A, Nepogodiev D, Doughty H, Bowley DM (2013). Meta-analysis of plasma to red blood cell ratios and mortality in massive blood transfusions for trauma. Injury, Int J Care Injured..

[3] Borgman MA, Spinella PC, Perkins JG, Grathwohl KW, Repine T, Beekley AC, et al. The ratio of blood products transfused affects mortality in patients receiving massive transfusions at a combat support hospital. J Trauma. 2007;63(4):805–13. 3. 10.1097/TA.0b013e3181271ba3 18090009

[4] Brohi K, Cohen MJ, Ross A (2007). Davenport. Acute coagulopathy of trauma: mechanism, identification and effect. Curr Opin Crit Care.

[5] Brohi K, Singh J, Heron M, Coats T (2003). Acute Traumatic Coagulopathy. J Trauma..

[6] Bulger EM, May S, Kerby JD, Emerson S, Stiell IG, Schreiber MA (2011). ROC Investigators. Out-of-hospital hypertonic resuscitation following traumatic hypovolemic shock: a randomized, placebo controlled trial. Ann Surg.

[7] Chow CC, Clermont G, Kumar R, Lagoa C, Tawadrous Z, Gallo D (2005). The acute inflammatory response in diverse shock states. Shock..

[8] DAMOCLES Study group (2005). A proposed charter for clinical trial data monitoring committees: helping them to do their job well. Lancet.

[9] Daniel‐Johnson J, Leitman S, Klein H, Alter H, Lee-Stroka A, Scheinberg P (2009). Probiotic-associated high-titer anti-B in a group A platelet donor as a cause of severe hemolytic transfusion reactions. Transfusion.

[10] Del Junco DJ, Holcomb JB, Fox EE, Brasel KJEA, Phelan HA, Bulger EM (2013). Resuscitate early with plasma and platelets or balance blood products gradually: Findings from the PROMMTT study. J Trauma Acute Care Surg..

[11] Dretzke J, Smith IM, James RH, Midwinter MJ (2014). Protocol for a systematic review of the clinical effectiveness of pre-hospital blood components compared to other resuscitative fluids in patients with major traumatic haemorrhage. Syst Rev..

[12] Farrington CP, Manning G (1990). Test statistics and sample size formulae for comparative binomial trials with null hypothesis of non-zero risk difference or non-unity relative risk. Stat Med..

[13] Frith D, Goslings JC, Gaarder C, Maegele M, Cohen MJ, Allard S (2010). Definition and drivers of acute traumatic coagulopathy: clinical and experimental investigations. J Thromb Haemost..

[14] Harris PA, Taylor R, Thielke R, Payne J, Gonzalez N, Conde JG (2009). Research electronic data capture (REDCap)–a metadata‐driven methodology and workflow process for providing translational research informatics support. J Biomed Informat.

[15] Hess JR, Brohi K, Dutton RP, Hauser CJ, Holcomb JB, Kluger Y (2008). The coagulopathy of trauma: a review of mechanisms. J Trauma..

[16] Ho AM, Dion PW, Yeung JH, Holcomb JB, Critchley LA, Ng CS (2012). Prevalence of survivor bias in observational studies on fresh frozen plasma:erythrocyte ratios in trauma requiring massive transfusion. Anesthesiology..

[17] Holcomb JB, Donathan DP, Cotton BA, Del Junco DJ, Brown G, Wenckstern TV, et al. Prehospital transfusion of plasma and red blood cells in trauma patients. Prehosp Emerg Care. Epub ahead of print 2014 Jun 16. 10.3109/10903127.2014.923077 24932734

[18] Holcomb JB, Wade CE, Michalek JE, Chisholm GB, Zarzabal LA, Schreiber MA (2008). Increased plasma and platelet to red blood cell ratios improves outcome in 466 massively transfused civilian trauma patients. Ann Surg..

[19] Karam O, Tucci M, Combescure C, Lacroix J, Rimensberger PC. Plasma transfusion strategies for critically ill patients. Cochrane Database of Systematic Reviews 2013, Issue 12. Art. No.: CD010654. doi:10.1002/14651858.CD010654.pub2. 10.1002/14651858.CD010654.pub2 PMC12001317 24374651

[20] Kauvar DS, Lefering R, Wade CE (2006). Impact of hemorrhage on trauma outcome: an overview of epidemiology, clinical presentations, and therapeutic considerations. J Trauma..

[21] Kim BD, Zielinski MD, Jenkins DH, Schiller HJ, Berns KS, Zietlow SP (2012). The effects of prehospital plasma on patients with injury: a prehospital plasma resuscitation. J Trauma Acute Care Surg.

[22] Maegele M, Lefering R, Yucel N, Tjardes T, Rixen D, Paffrath T (2007). Polytrauma of the German Trauma Society (DGU) Early coagulopathy in multiple injury: an analysis from the German Trauma Registry on 8724 patients. Injury..

[23] Marshall JC (2001). Inflammation, coagulopathy, and the pathogenesis of multiple organ dysfunction syndrome. Crit Care Med..

[24] Narick C, Triulzi DJ, Yazer MH (2012). Transfusion-associated circulatory overload after plasma transfusion. Transfusion.

[25] Schulz KF, Altman DG, Moher D, for the CONSORT Group. CONSORT 2010 Statement: updated guidelines for reporting parallel group randomised trials. BMJ 2010;340:c332 doi:10.1136/bmj.c332. 10.1136/bmj.c332 PMC2844940 20332509

[26] van Stein D, Beckers EA, Sintnicolaas K, Porcelijn L, Danovic F, Wollersheim JA (2010). Transfusion-related acute lung injury reports in the Netherlands: an observational study. Transfusion.

[27] Watson GA, Sperry JL, Rosengart MR, Minei JP, Harbrecht BG, Moore EE (2009). Inflammation and Host Response to Injury Investigators: Fresh frozen plasma is independently associated with a higher risk of multiple organ failure and acute respiratory distress syndrome. J Trauma.

[28] Whitaker BI, Hinkins S. The 2011 National Blood Collection and Utilization Survey Report. United States Department of Health and Human Services, Washington, D.C. 87 pp. http://www.hhs.gov/ash/bloodsafety/nbcus/

[29] Wo CJ, Shoemaker WC, Appel PL, Bishop MH, Kram HB, Hardin E (1993). Unreliability of blood pressure and heart rate to evaluate cardiac output in emergency resuscitation and critical illness. Crit Care Med..

